# Disentangling Depression and Distress Networks in the Tinnitus Brain

**DOI:** 10.1371/journal.pone.0040544

**Published:** 2012-07-12

**Authors:** Kathleen Joos, Sven Vanneste, Dirk De Ridder

**Affiliations:** Brai^2^n, Tinnitus Research Initiative Clinic Antwerp, Department of Neurosurgery, University Hospital Antwerp, Belgium; University of Regensburg, Germany

## Abstract

Tinnitus is the continuous perception of an internal auditory stimulus. This permanent sound often affects a person's emotional state inducing distress and depressive feelings changes in 6–25% of the affected population. Distress and depression are two distinct emotional states. Whereas distress describes a transient aversive state, interfering with a person's ability to adequately adapt to stressors, depressive feelings should rather be considered as a more constant emotional state. Based on previous observations in chronic pain, posttraumatic stress disorder and depression, we assume that both states are related to separate neural circuits. We used the Dutch version of the Tinnitus Questionnaire to assess the global index of distress together with the Beck Depression Inventory to evaluate the depressive symptoms accompanying tinnitus. Furthermore sLORETA analysis was performed to correlate current density distribution with distress and depression scores, revealing a lateralization effect of depression versus distress. Distress is mainly correlated with alpha 2, beta 1 and beta 2 activity of the right frontopolar cortex and orbitofrontal cortex in combination with beta 2 activation of the anterior cingulate cortex. In contrast, the more permanent depressive alterations induced by tinnitus are associated with activity of alpha 2 activity in the left frontopolar and orbitofrontal cortex. These specific neural circuits are embedded in a greater neural network, with the parahippocampal region functioning as a crucial linkage between both tinnitus related pathways.

## Introduction

Tinnitus is the awareness of a tone, a ringing or buzzing sound in the absence of an external auditory stimulus, also defined as a phantom phenomenon [1]. The constant perception of this internal sound frequently causes a considerable amount of distress. It has a prevalence of up to 10%–15% in an adult population, with an increasing prevalence with increasing age [2]–[4]. About 6% to 25% of tinnitus patients report that their quality of life is reduced [2], [5], [6], with 2–4% of the total population suffering in the worst degree [3]. Thus, although a considerable amount of people experience tinnitus, 1 out of 5 is emotionally seriously affected by it reporting sleep disturbances, lack of energy and mood disorders [7]. There is no correlation between the amount of distress and the perceived loudness as measured by tinnitus matching [1], suggesting that two separate neural networks might encode tinnitus intensity and tinnitus distress.

Tinnitus can be considered an auditory phantom phenomenon [8] similar to deafferentation pain seen in the somatosensory system [9]–[11], related to reorganization [12], [13] and hyperactivity [14], [15] of the auditory central nervous system. New insights into the neurobiology of tinnitus suggest that neuronal changes are not limited to the classical auditory pathways. In particular, the insula [16], anterior cingulate cortex (ACC) [17], [18], amygdala, dorsolateral prefrontal cortex [19] and (para)hippocampus (PHC) [20]–[22] seem to play a specific role in tinnitus as they take part in the neural circuit underlying tinnitus related distress.

Studying the affective dimension of pain and tinnitus, distress has been considered an aversive state in which a person is unable to adapt completely to stressors (i.e. pain or tinnitus) [23], [24]. The brain areas involved in tinnitus related distress are also involved in the emotional component of the pain matrix such as the ACC, prefrontal cortex, amygdala, and insula [25]–[29].

However apart from distress, studies indicate that depressive feelings are an important aspect in pain and tinnitus as well. About 40% of tinnitus patients report to suffer from mood disorders such as depression due to their tinnitus [30]. Mood has been defined as a relatively long lasting emotional state that is less specific, less intense, and less triggered by a particular event [23], [24]. As such, mood can be seen as a stable long-term change induced by the persistence of pain and pain distress [29] and depression is a pathological mood state. In pain, the underlying emotional network related to mood or depressive feelings comprises the medial prefrontal cortex, amygdala, PHC, insula and ACC [25], [31], [32], emphasizing the crucial role of the frontal brain regions, which have been associated with the secondary affect in chronic pain [29].

Chronic pain and tinnitus show many similarities in symptoms [33] and pathophysiology [34], [35]. For example, a touch stimulus to the skin can evoke a painful sensation (allodynia) in patients with chronic pain, while tinnitus patients often perceive specific sounds as unpleasant or painful (misophonia) [36]. The generation of these characteristic symptoms is due to a wind-up phenomenon caused by neural plasticity. Furthermore in chronic pain and tinnitus brain areas beyond the somatosensory and auditory brain regions are involved [37]. Furthermoreelectrical stimulation of these sensory cortices has the ability of relieving or masking both pain and tinnitus [38]. Based on the various similarities between pain and tinnitus, we expect that, in analogy to chronic pain [29], distress and depression are generated by distinct neural networks. Hence, the main goal of this study is to disentangle the networks related to distress and depressive feelings caused by tinnitus and to separate these from the observed neural changes associated with the perception of the tinnitus sound using source analysis of the resting-state EEG activity (eyes closed). Depressive feelings are assessed by the Dutch version of the Beck Depression Inventory-II (BDI-II) and the tinnitus related distress by using the Tinnitus Questionnaire (TQ) and correlated to continuous scalp EEG recordings and Low Resolution Electromagnetic Tomography (sLORETA), a tomographic inverse solution imaging technique.

## Results

### Behavior measures

A significant correlation was obtained between BDI and TQ r = .29, *p*<.05. In addition a significant correlation was obtained between TQ and distress as measured with the numeric rating scale (NRS) (r = .41, *p*<.0). No significant effect was found between the TQ and respectively age and tinnitus duration, nor was there an association with tinnitus laterality and tinnitus type. A similar analysis with the BDI and distress as measured by the NRS, age, tinnitus duration, tinnitus laterality and tinnitus type yielded no significant effect.

### Source localization BDI

**Figure 1:**
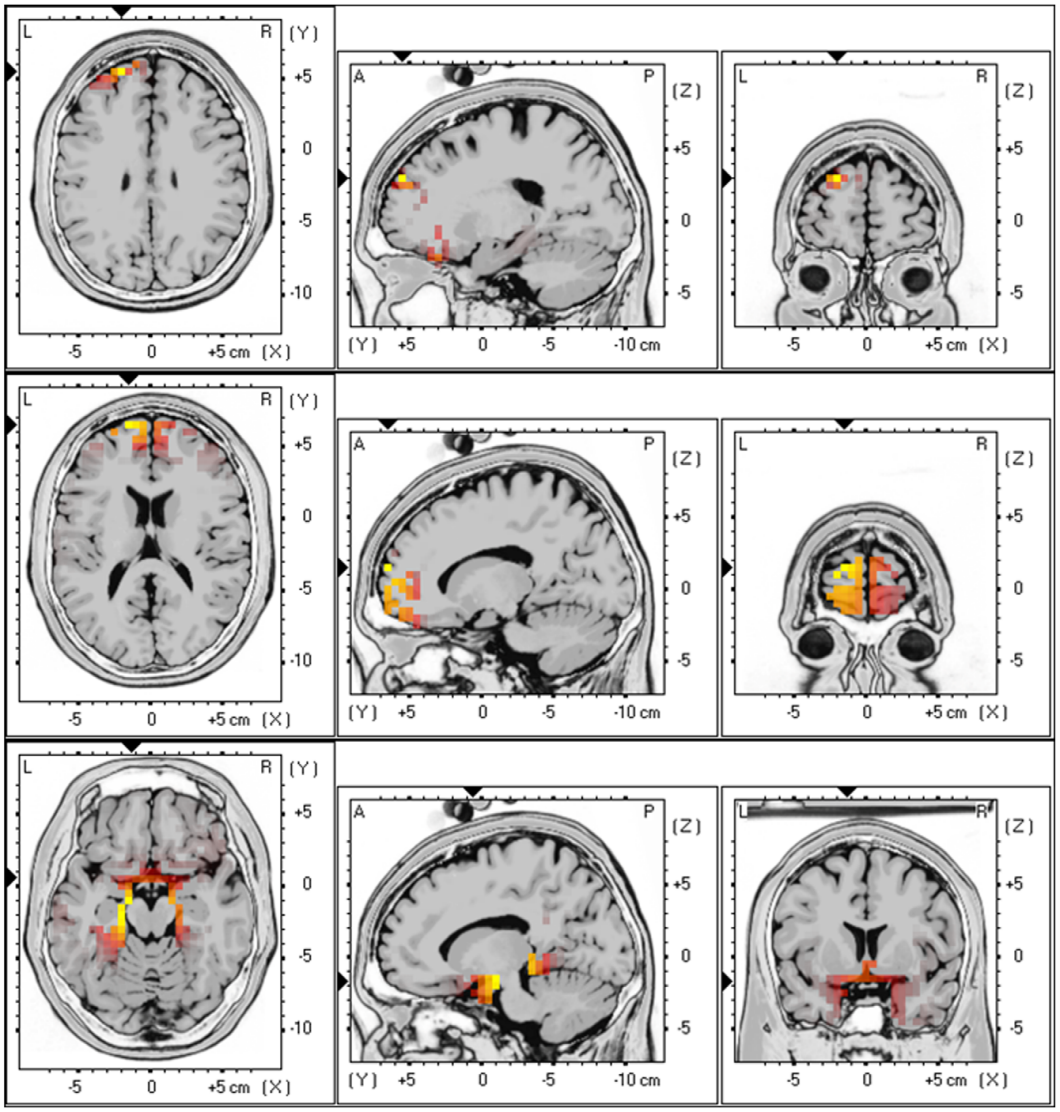
Correlation between the Beck Depression scale and respectively alpha 1 (r = .34) and alpha 2 (r = .37) activity in the frontopolar and orbitofrontal cortex (BA10 & BA11) and beta 3 (r = .27) activity in the subgenual anterior cingulate cortex (BA25) and parahippocampal area (BA35 & 36).

Analyzing the results with sLORETA we observed a significant positive correlation (*p*<.05) between BDI scores and alpha 1 (r = .34, *p*<.05) and alpha 2 (r = .37, *p*<.01) activity in the frontopolar and orbitofrontal cortex (OFC) (Brodmann area (BA) 10 and BA11) and beta 3 (r = .27, *p*<.05) activity in the sgACC (BA25) and the PHC (BA35&36) (see figure 1). No significant correlations could be detected between depression severity, assessed by the BDI-II, and other frequency bands.

#### Source localization

**Figure 2:**
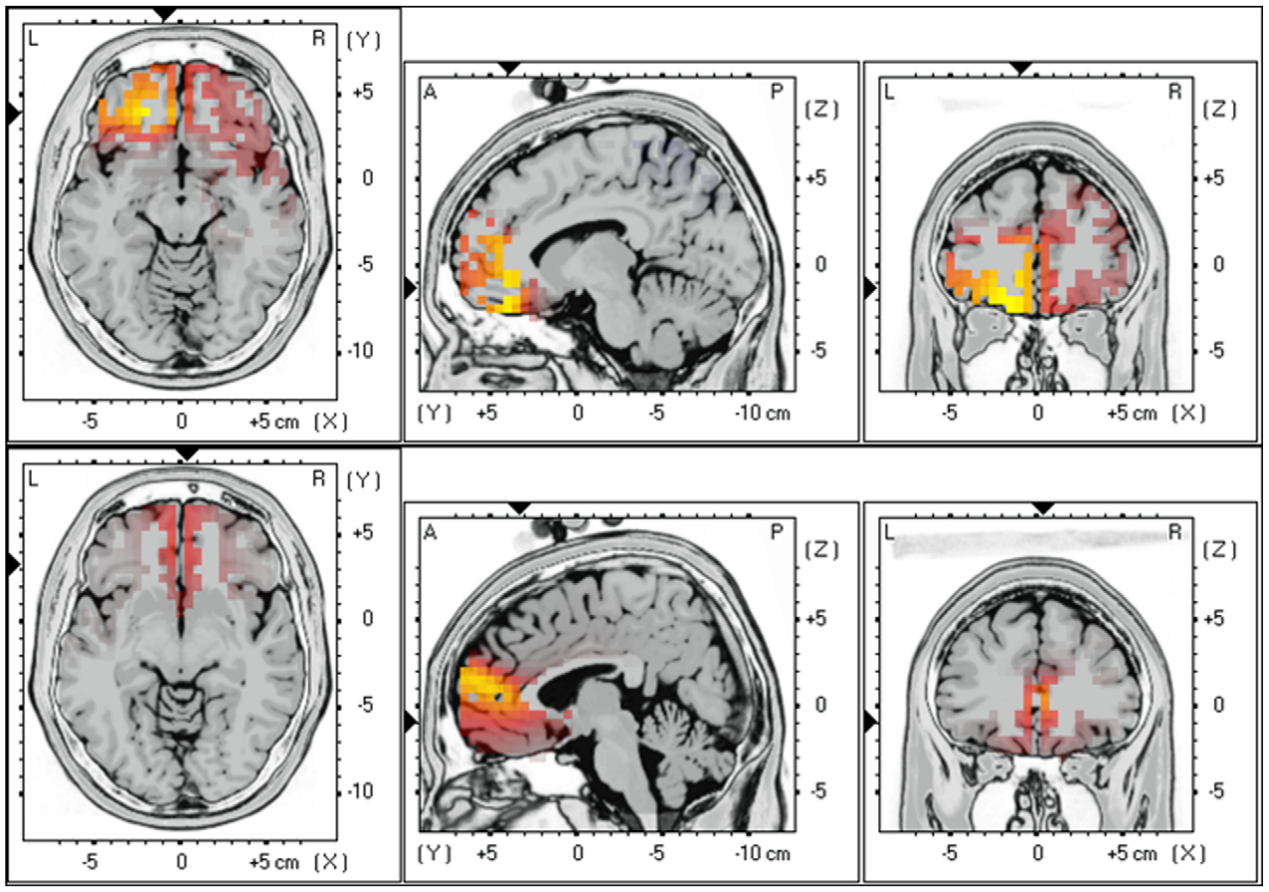
Correlation between the Tinnitus questionnaire and respectively alpha 2 (r = .30) and beta 2 (r = .29) activity in the frontopolar and OFC (BA10, BA11), subgenual anterior cingulate cortex (BA25), pregenual anterior cingulate cortex (BA24).

*TQ*A sLORETA analysis demonstrated a significant positive correlation between TQ scores and alpha 2 (r = .30, *p*<.05) and beta 2 (r = .29, *p*<.05) activity in the frontopolar and OFC (BA10, BA11), subgenual ACC (sgACC)(BA25) and ACC (BA24) (see figure 2). No significant correlations could be detected between the TQ and other frequency bands.

### Source localization of distress as measured by the numeric rating scale

**Figure 3:**
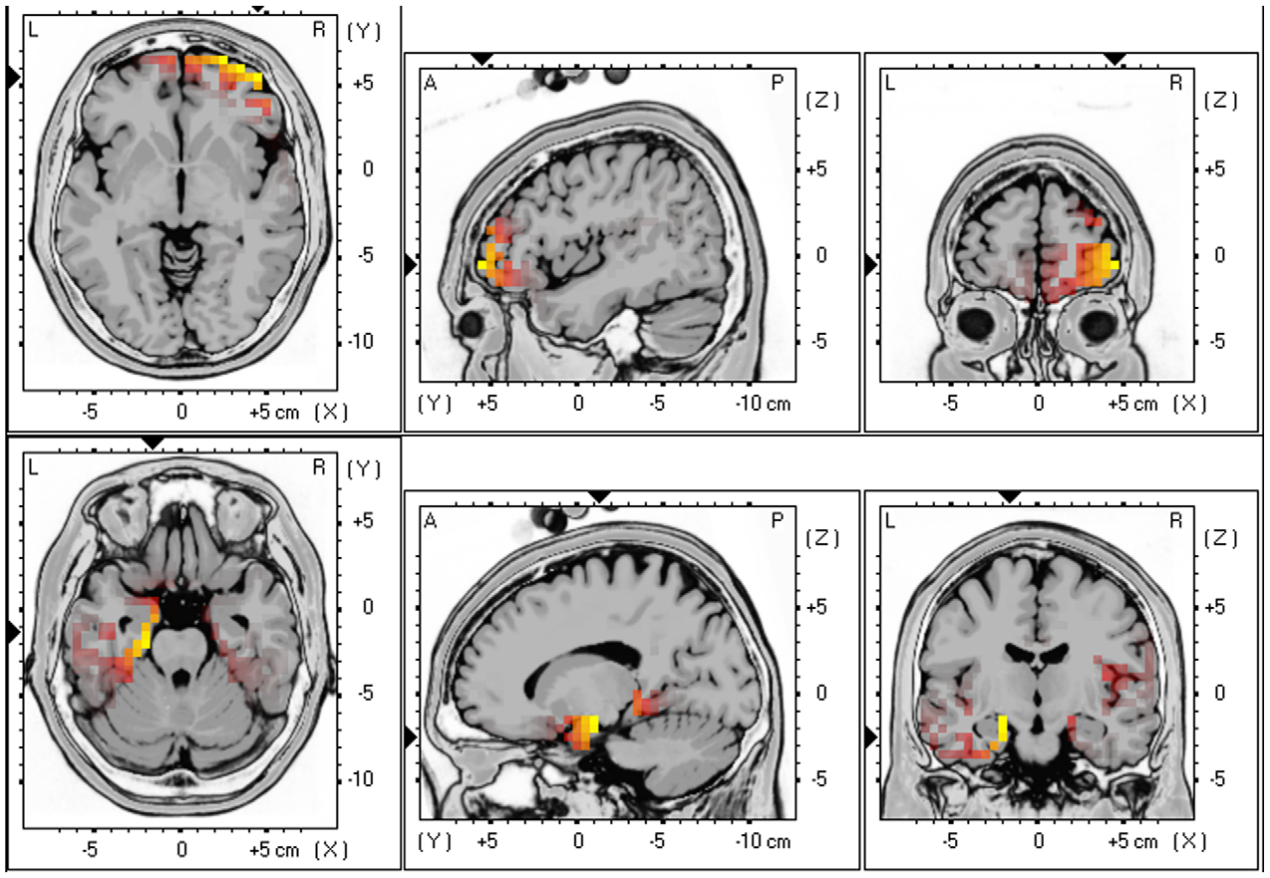
Correlation between the distress using a numeric rating scale and respectively alpha 2 (r = .34) and beta 3 (r = .40) activity in the frontopolar and OFC (BA10, BA11), subgenual anterior cingulate cortex (BA25), and and parahippocampal area (BA27 & 28).

sLORETA correlation demonstrated a significant positive effect between the distress using a NRS and respectively alpha 2 activity (r = .34, *p*<.05) in the frontopolar and OFC (BA10, BA11) and beta 3 activity (r = .40, *p*<.05) subgenual anterior cingulate cortex (BA25), and and parahippocampal area (BA27 & 28) (see figure 3). No significant correlations could be detected between the distress using a NRS and other frequency bands.

### Region of interest analysis

The BDI correlates significantly with left and right frontopolar and OFC (BA10 & BA11), the ACC (BA24), and the sgACC (BA25) for the alpha 2 frequency band (see Table 1). By conducting a partial correlation analysis for BDI, controlling for TQ, a significant correlation was only obtained for the left frontopolar and OFC (BA10 & BA11). For beta 3 a significant positive correlation was found between the left and right PHC (BA35&36). However, when controlling for the TQ this effect did not hold. By conducting a partial correlation analysis for BDI, controlling for distress using a NRS, a significant correlation was only obtained for the left frontopolar and OFC (BA10 & BA11). No correlations could be found for other frequency bands, or with other regions of interest.

**Table 1:** Significant correlations and partial correlations for the BDI and TQ and regions of interest.

			BDI	TQ	BDI controlled for TQ	TQ controlled for BDI
Alpha2	BA10 Left	OFC	.38**	.37**	.28*	.24
	BA10 Right	OFC	.32**	.35**	.21	.26†
	BA11 Left	OFC	.37**	.37**	.28*	.24
	BA11 Right	OFC	.36**	.40**	.23	.30**
	BA24	ACC	.29*	.15	.20	.22
	BA25	sgACC	.28*	.31**	.20	.17
Beta1	BA10 Right	OFC	.17	.30**	.06	.26†
	BA11 Right	OFC	.19	.33**	.07	.28*
Beta2	BA10 Right	OFC	.18	.37**	.04	.33**
	BA11 Right	OFC	.20	.43**	.07	.39**
	BA24	ACC	.22	.29*	.13	.27*
	BA25	sgACC	.19	.35**	.05	.31**

† *p*<.10; *p<.05; **p<.01.

For the TQ a significant positive correlation was found between left and right frontopolar and OFC (BA10 & BA11) and the sgACC (BA25) for the alpha 2 frequency band. For the alpha 2 frequency band only a significant positive partial correlation could be found between the right OFC (BA11) and TQ, controlling for BDI, although there is a trend to significance between the TQ and the right frontopolar cortex (BA10). For the beta 1 frequency band a significant positive correlation was found between the right frontopolar and OFC (BA10 & 11) and the TQ. Although only a significant partial correlation could be obtained between the right OFC (BA11) and TQ when controlling for BDI, with a trend to significance for the right frontopolar cortex and the TQ. As for beta 2 significant partial correlations could be obtained between the right frontopolar and OFC (BA10 & 11), the ACC (BA24) and the sgACC (BA25) and TQ.

For distress using a NRS a significant positive correlation was found between left and right frontopolar and OFC (BA10 & BA11) for the alpha 2 frequency band (see Table 2). For the alpha 2 frequency band only a significant positive partial correlation could be found between the right OFC (BA10 & BA11) and distress using a NRS, controlling for BDI. For the beta 3 frequency band a significant positive correlation was obtained for ACC (BA24) and sgACC, this effect remained after controlling for BDI. No correlations could be found for other frequency bands, or with other regions of interest.

**Table 2:** Significant correlations for distress using a numeric rating scale and BDI for specific regions of interest.

			BDI	Distress	BDI controlled for Distress	Distress controlled for BDI
Alpha2	BA10 Left	OFC	.38**	.27*	.36**	.25
	BA10 Right	OFC	.32**	.34*	.22	.29*
	BA11 Left	OFC	.37**	.25	.35**	.23
	BA11 Right	OFC	.36**	.37**	.20	.34**
	BA24	ACC	.29*	.22	.19	.25
	BA25	sgACC	.28*	.27*	.16	.23
Beta 3	BA24	ACC	.22	.32*	.15	.31**
	BA25	sgACC	.19	.36**	.12	.35**

* *p*<.05; **p<.01.

## Discussion

In this study we focused on the differences between the neural circuits underlying depression and tinnitus-related distress.

A significant correlation was obtained between the BDI and TQ indicated that both questionnaires have some relation. However this correlation was rather weak explaining only .08% of the variance, indicating that both questionnaires e only partly overlie each other, but that they measure other concepts.

Using the TQ to assess the severity of perceived tinnitus related distress, a positive correlation was demonstrated for the frontopolar, OFC and sgACC in the alpha 2 band together with a positive correlation of beta 1 and beta 2 activity in the right frontopolar and OFC cortex. In addition a positive correlation is present with the beta 2 activity in the pgACC and sgACC. These findings indicate that the higher score for tinnitus related distress goes together with increased activity in the frontopolar, OFC, sgACC and pgACC. Partial correlations further demonstrated more precisely that tinnitus related distress correlates exclusively with the right frontopolar and OFC for alpha 2, beta 1 and beta 2, as well as with the ACC and sgACC.

Using the BDI to measure severity of depression, positive correlations could be revealed between BDI-II and respectively alpha 1 and alpha 2 in the frontopolar and OFC, and beta 3 activity in the sgACC, indicating that patients with higher scores on the BDI-II showed an increased synchronized activity in frontopolar and OFC, and sgACC. Partial correlations further demonstrated that depressive feelings exclusively correlates with the left frontopolar and OFC in alpha 2, showing that the higher a patient scores on depressive feelings the more current density is demonstrated in the left OFC for alpha 2. For these latter results this could be replaced using distress as measured by a NRS. Again is was shown that depressive feelings exclusively correlates with the right frontopolar and OFC in alpha 2 and distress exclusively correlates with the right frontopolar and OFC in alpha 2.

### Distress

The neural correlates underlying distress have been explored in different pathologies. One of the most explored fields is distress associated with chronic pain. The ACC has been verified to play a pivotal role in the awareness of pain unpleasantness [29], [39]. This region is associated with a large array of functions such as attention, analysis of sensory information, behavior, emotions and regulation of visceral functions. Moreover altered activity of the ACC can lead to abnormal emotional behavior and might contribute to the depressive symptoms and negative affect [29], [39] caused by tinnitus. Previous studies using voxel-based morphometry [40], [41], fMRI [42] and positron emission tomography [43] identified the ACC as a critical component of the emotion-processing network underlying the pathophysiology of mood disorders. This is in accordance to findings in this study, namely a significant positive correlation with the TQ in the beta 2 frequency band.

Apart from the pregenual ACC, the sgACC showed an increase in alpha 2 and beta 2 frequencies in correlation with TQ scores and increase in alpha 2 and beta 3 in correlation with the NRS measuring distress. Neuroimaging techniques confirmed the critical role of the sgACC in depressive [44], [45] and posttraumatic stress disorder [46]. The sgACC has also been associated with storing negatively valenced memories [47], processing of aversive sounds [48] and unpleasant music [49] as well as tinnitus [50] and tinnitus related distress [21]. Severe distress, such as in posttraumatic stress disorder, has already been associated with increased beta activity especially over frontal and central areas [51], [52]. In control subjects the dorsal part of the ACC and the prefrontal cortex generate frontal midline theta (alternating with the VMPFC) [53]. In distressed tinnitus patients in contrast to controls alpha and beta activity is increased [21]. In highly distressed versus lowly distressed patients alpha is even more increased, suggesting that the amount of alpha activity correlates with the amount of distress, while it has been hypothesized that beta activity might represent a more general distress activity, compatible with what is known for post-traumatic stress disorders [51], [52]. Additionally, based on the findings reported here, it can be assumed that tinnitus related distress mainly correlates with right frontopolar and OFC alpha and beta activity in contrast to more long-standing depressive changes induced by tinnitus.

### Depressive feelings

Depressive symptoms are extremely heterogeneous and it is impossible to link these complex neurobehavioral changes to one specific brain structure. Depression can be seen as a dysfunctional limbic-cortical network complementary to failure of intrinsic compensation of the remaining circuit to preserve homeostatic emotional control in stressful situations. An interesting model has been proposed that consists of 7 brain regions, consistently identified by previous studies. Those areas include the anterior thalamus, hippocampus, dorsal prefrontal, medial frontal, OFC, ACC and sgACC [54]. Especially a decreased frontal lobe function, including the frontopolar and OFC (i.e. BA 10 and 11), is consistently identified to play an important contributing factor.

The OFC is not only involved in the pathophysiology of depression [55], [56] but also in the storage of implicitly acquired linkages between factual knowledge and bio-regulatory stores, including those that constitute feelings and emotions [57]. Another interesting observation is that this brain area is implicated in the emotional processing of sound [49], [58]–[60]. For example, patients with OFC lesions had reduced self-evaluated perception of the unpleasantness of an acoustic probe stimulus [61].

### Lateralization of depression and distress

**Figure 4:**
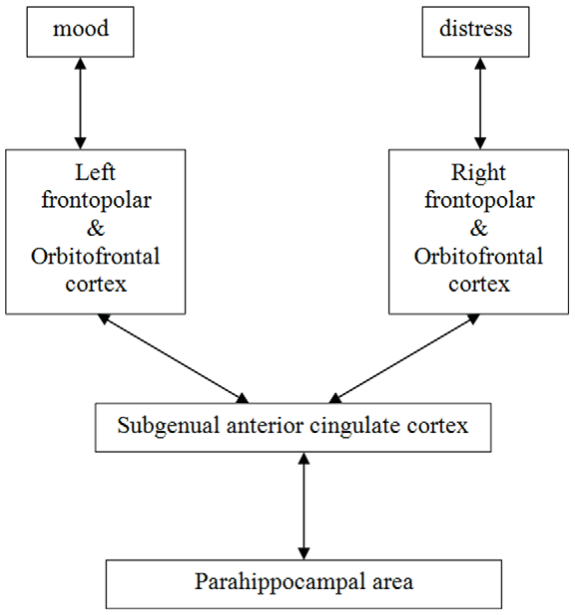
A hypothetical model based on our findings.

Distress and depression are two different components of affective disturbances. Whereas depressive feelings are mainly localized to the left frontal side, distress is mostly linked to a change of right frontal brain activity (see Figure 4). This assumption, based on our current results, confirms previous findings in which increased alpha power was related to frontal lateralisation in depression and depressive feelings [62], [63]. Moreover, recent studies provide evidence that there is a clear relationship between EEG activity and depressive feelings with a frontal asymmetry often based on the alpha frequency range [64], [65]. More evidence to prove this hypothesis can be found in prior research on transcranial direct current stimulation (tDCS), which is a non-invasive and painless device that modulates cortical excitability in the brain regions of interest through a weak direct electrical current. Depending on the polarity of the stimulation, tDCS can increase or decrease cortical excitability in the brain regions to which it is applied [66]. Using anodal tDCS to the left frontal brain is effective in reducing depressive symptoms [67]. Left anodal stimulation might be successful because it has the ability of restoring hypoactivity of the left prefrontal cortex, further correcting the imbalance between left and right hemisphere, that leads to mood disturbances [68], [69].

In contrast to the importance of the left frontopolar and OFC in depressive feelings alterations, distress is mainly generated by the right frontopolar and OFC. For example, bilateral frontal tDCS, placing the anode right and the cathode left, was able to reduce tinnitus intensity and distress although switching the poles couldn't show any significant effect [70].

In addition to the side specificity, depressive feelings were mainly determined by the presence of alpha 2 activity, while distress correlated more with beta 1 and beta 2 frequency in addition to its correlation with the alpha 2 frequency range. Taking these findings together the frontopolar and OFC differentiates the affective component of tinnitus in depressive and distress changes.

### Parahippocampal area

Another area, namely the PHC, is most likely implicated in the depressive alterations provoked by the phantom sound. A significant increase in beta 3 activity at the PHC was identified using source localization in patients with higher scores on the BDI-II. In addition, based on a region of interest analysis a significant positive correlation was found between the left and right PHC (BA35) and the BDI. However, when controlling for the TQ this effect did not persist, suggesting that the PHC is important in both tinnitus related distress and depressive feelings.

The PHC together with the auditory and prefrontal cortex, constitutes a network associated with auditory sensory gating [71]–[74], suppressing irrelevant or abundant noise [75]. It has been hypothesized that the hippocampal region is involved in constantly updating the tinnitus, consequently avoiding habituation of the tinnitus percept [20]. This is based on supraselective amytal testing via the anterior choroidal artery which supplies the amygdalohippocampal area, resulting in transient inactivation of the amygdalohippocampal region. This inactivation has the capability of suppressing tinnitus intensity [20]. Hence, the parahippocampal gyrus may play a crucial role in the presence of tinnitus and has the ability to prevent the tinnitus percept to be constantly updated or to be extracted from the hippocampal memory. Similar to the OFC, activation of the (para)hippocampal region has been observed while listening to unpleasant music during functional magnetic resonance imaging (fMRI) [76]. Considering this, together with the knowledge of parahippocampal connectivity to the sgACC [77] and OFC [78], we assume that the parahippocampal gyrus might be a decisive link between the tinnitus related network and the attention-emotional circuit underlying tinnitus-related emotional disturbances.

### Limitations of the study

One major limitation of this and any EEG based approach is that no subcortical activity can be analyzed, limiting network description to cortical sources. The data presented should therefore be viewed acknowledging this limitation.

Depression and distress are related to each other. It is however stated that when there is a low multicollinearity (the correlation among independent variables) it is possible to apply partial correlations [79], [80]. As our analysis showed there was only a small correlation between depressive feelings and distress, it is permitted to apply partial correlation analysis. In addition our results were replicated using a NRS measuring distress, and this NRS did not correlate with depression.

### Conclusion

In conclusion, tinnitus related distress and more chronic changes in depressive feelings are associated with specific alterations in brain activity of separate neural pathways. We assume that both emotional aspects have their own specific neural circuit embedded within a larger common network. The network responsible for distress is mainly correlated with beta 1 and beta 2 activity of the right frontopolar and OFC, as well as beta 2 frequency in the ACC. The continuous awareness of tinnitus accompanied by distress can induce more long-term changes in depressive feelings. This more constant emotional disturbance, assessed by the BDI-II, can be linked to alpha 2 synchronized activity in the left frontopolar and OFC. Furthermore we assume that the parahippocampal area may be a crucial connection between the tinnitus-related network and the emotion related neural pathways.

## Methods

### Participants

The patient group included fifty-six patients (N = 56; 27 males and 29 females) with narrow band noise tinnitus, with a mean age of 54.74 (SD  = 14.49). As no differences in emotional state nor distress have been demonstrated related to uni- or bilaterality [81] both groups are included in the study. Thirty-six patients have bilateral tinnitus and twenty have unilateral tinnitus. The mean tinnitus duration was 6.92 years (SD  = 9.51). Individuals with pulsatile tinnitus, Ménière's disease, otosclerosis, chronic headache, neurological disorders such as brain tumors, and individuals being treated for mental disorders were excluded from the study in order to obtain a more homogeneous sample. To obtain an even more uniform population we only selected patients with narrow band noise. All patients were investigated for the extent of hearing loss using audiograms. Tinnitus patients were tested for the frequency and the minimum masking level of their tinnitus. They were interviewed as to their perceived location of the tinnitus (exclusively in the left ear, predominantly in the left ear, in both ears, and centralized in the middle of the head (bilateral), predominantly in the right ear, exclusively in the right ear).

To assess the severity of depression, a chronic pathological negative mood state that frequently accompanies tinnitus, all patients filled out the validated Dutch version of the Beck Depression Inventory-II (BDI-II) [82] originally published in 1961 by Beck et al. This questionnaire was revised in 1996 (BDI-II) following the DSM-IV symptom criteria [83]. The BDI-II is a self-report inventory consisting of 21 items evaluating depressive feelings during the last week. The severity of the depressive feelings is attained by assigning each item with a score ranging between 0–4. Based on the total score of the BDI, patients are categorized in: no or minimal depression (0–9); rand (10–14); mild (15–20); moderate (21–30); severe (31–40); very severe (41–63) depression. The mean BDI score was 10.95 (SD  = 9.73).

Patients were also given the validated Dutch version of the Tinnitus Questionnaire [84], [85] originally published by Goebel and Hiller [86]. Goebel and Hiller described this TQ as a global index of distress and the Dutch version was further confirmed as a reliable measure for tinnitus-related distress [85]. Based on the total score on the TQ, participants were assigned to a distress category: slight (0–30 points; grade 1), moderate (31–46; grade 2), severe (47–59; grade 3), and very severe (60–84; grade 4) distress. Furthermore, Goebel and Hiller (1994) stated that grade 4 tinnitus patients are psychologically decompensated, indicating that patients categorized into this group cannot cope with their tinnitus. In contrast, patients that have a score lower than 60 on the TQ can cope with their tinnitus. The mean TQ score was 40.93 (SD  = 17.03).

Lastly, patients also filled out a NRS measuring distress (“How stressful is your tinnitus? 0  =  no distress and 10 =  suicidal distress”).

The study was approved by the Ethical Committee of the Antwerp University Hospital, Belgium. Patients signed an informed consent.

### EEG data collection

EEGs (Mitsar, Nova Tech EEG, Inc, Mesa) were obtained in a fully lighted room with each participants sitting upright in a comfortable chair. The EEG was sampled with 19 electrodes (Fp1, Fp2, F7, F3, Fz, F4, F8, T7, C3, Cz, C4, T8, P7, P3, Pz, P4, P8, O1 O2) in the standard 10–20 international placement referenced to linked lobes and impedances were checked to remain below 5 kΩ. Data were collected for 100 2-s epochs eyes closed, sampling rate  = 1024 Hz, and band passed 0.15–200 Hz. Data were resampled to 128 Hz, band-pass filtered (fast Fourier transform filter applying a Hanning window) to 2–44 Hz. These data were transposed into Eureka! Software [87], plotted and carefully inspected for manual for artifact. All episodic artifacts including eye blinks, eye movements, teeth clenching, body movement, or ECG artifacts were removed from the stream of the EEG. In addition, an independent component analysis (ICA) was conducted to further verify if all artifacts were excluded. To investigate the effect possible ICA component rejection we compared the power spectra in two approaches: (1) after visual artifact rejection only (before ICA) and (2) after additional ICA component rejection (after ICA). To test for significant differences between the two approaches we performed a repeated-measure ANOVA, considering mean band power as within-subject variables and groups (unilateral vs. bilateral tinnitus) as between-subject variable. The mean power in delta (2–3.5 Hz), theta (4–7.5 Hz), alpha1 (8–10 Hz), alpha2 (10–12 Hz), beta1 (13–18 Hz), beta2 (18.5–21 Hz), beta3 (21.5–30 Hz) and gamma (30.5–45 Hz) did not show a statistically significant difference between the two approaches. Therefore, we continued by reporting the results of ICA corrected data.

### Source Localization

Standardized low-resolution brain electromagnetic tomography (sLORETA) was used to estimate the intracerebral electrical sources that generated the scalp-recorded activity in each of the eight frequency bands [88]. sLORETA computes electric neuronal activity as current density (A/m^2^) without assuming a predefined number of active sources, giving us a global idea of the electrical activity of neuronal cell assemblies with a high temporal resolution. The sLORETA solution space consists of 6239 voxels (voxel size: 5×5×5 mm) and is restricted to cortical gray matter and hippocampi, as defined by digitized MNI brain are derived from the international 10/20 system [89].

### Region of interest analysis

The log-transformed electrical current density was averaged across all voxels belonging to the region of interest, Brodmann areas selected on previous research, respectively frontopolar cortex (BA10) [90] left and right separately, OFC (BA11) [90] left and right separately, ACC (BA24; left and right combined) [29], and subgenual ACC (sgACC; BA25; left and right combined) [21] separately for frequency bands delta (2–3,5 Hz), theta (4–7,5 Hz), alpha 1 (8–10 Hz), alpha2 (10,5–12,5 Hz), beta1 (13–18 Hz), beta2 (18.5–21 Hz), beta3 (21.5–30 Hz) and gamma (30.5–45 Hz).

### Statistical analyses

For the source localization we used a non-parametric methodology. It is based on estimating, via randomization (i.e. 5000), the empirical probability distribution for the max-statistic, under the null hypothesis. This methodology corrects for multiple testing (i.e., for the collection of tests performed for all voxels, and for all frequency bands). As explained by Nichols and Holmes, the SnPM methodology does not require any assumption of Gaussianity and corrects for all multiple comparisons [91].

In addition, for the region of interest analysis we conducted Pearson correlations and Partial correlations. Partial correlations measure the degree of relationship between two variables, with the effect of a set of controlling variables removed. In our cases we will correlate a region of interest for a certain frequency band with BDI, controlling for TQ and vice versa, thus obtaining source analyzed current density specifically for distress and depressive feelings separately.
